# Association between dietary behaviors and depression in adolescent girls

**DOI:** 10.1186/s12889-022-13584-0

**Published:** 2022-06-11

**Authors:** Abbas Ali Sangouni, Sara Beigrezaei, Shahab Akbarian, Majid Ghayour-Mobarhan, Emad Yuzbashian, Amin Salehi-Abargouei, Gordon A. Ferns, Sayyed Saeid Khayyatzadeh

**Affiliations:** 1 Nutrition and Food Security Research Center, Shahid Sadoughi University of Medical Sciences, Yazd, Iran; 2 Department of Nutrition, School of Public Health, Shahid Sadoughi University of Medical Sciences, Yazd, 8914715645 Iran; 3 International UNESCO center for health related basic sciences and human nutrition, department of nutrition, faculty of medicine, 37552Mashhad University of medical sciences, Mashhad, Iran; 4 Department of Agricultural, Food and Nutritional Sciences, University of Alberta, Edmonton, Alberta Canada; 5 Brighton & Sussex Medical School, Division of Medical Education, Falmer, Brighton, Sussex, BN1 9PH UK

**Keywords:** Dietary behaviors, Depression, Adolescent, Chewing, Snack, Spicy, Fluid

## Abstract

**Background:**

The growing prevalence of depression has become a major public health problem. There is limited evidence regarding the relationship between dietary behaviors and depression. The present study was designed to evaluate the association between dietary behaviors and depression score.

**Methods:**

A total of 933 Iranian adolescent girls aged 12 to 18 years were included in this cross-sectional study. Depression severity score was assessed using a validated Persian version of Beck’s depression inventory. Dietary behaviors were pre-defined and assessed in ten domains using a standard questionnaire. To investigate the association between dietary behaviors and depression score, the linear regression analysis in crude and adjusted models was used.

**Results:**

67.7% of participants had no or minimal depression symptoms and 32.3% of participants were categorized with mild-to-severe depression symptoms. There were significant inverse relationships between main meal consumption (Beta: -0.141; 95% CI: − 3.644 to − 1.000; *P = 0.001*), snack consumption (Beta: -0.100; 95% CI: − 2.400 to − 0.317; *P = 0.002*), regular meal consumption (Beta: 0.23; 95% CI: 0.13–0.42; *P = 0.001*) and food chewing (Beta: -0.152; 95% CI: − 2.279 to − 0.753; *P = 0.03*) with depression score. These associations remained significant after adjustment for confounding variables. In addition, frequency of intra-meal fluid intake (Beta: 0.096; 95% CI: 0.288 to 1.535; *P = 0.004*) and spicy foods consumption (Beta: 0.076; 95% CI: 0.098 to 1.508; *P = 0.02*) were directly associated with depression score in the crude model. These significant relations were disappeared in full adjusted model. No significant association was found between breakfast consumption, intake of fried foods, chewing ability, and tooth loss with depression score (*P > 0.05*).

**Conclusions:**

Significant associations were observed between specific eating behaviors with depression score. Prospective studies are needed to confirm these findings.

## Introduction

The increased prevalence of depression is a major public health problem that has imposed significant economic and health costs on governments [1, 2]. Depressed mood and anhedonia (loss of interest in daily activities) are two main symptoms of depression, and are mostly accompanied by other symptoms including change in weight or appetite, insomnia or hypersomnia, and worthlessness or guilt [3]. The global prevalence of depression is between 11.1–14.6% in the general population [4]. A meta-analysis demonstrated that the prevalence of depression diagnosed by the Beck Depression Inventory (BDI) is 43.5% in Iranian children and adolescents [5]. Notably, its prevalence is higher among girls [5]. Although the etiology of depression is not fully clear, the evidence suggested that inflammation and oxidative stress are linked to the pathogenesis of depression [6–9]. Investigations showed that depression could decrease quality of life [10, 11], and has reported as the most important cause of suicide in adolescents [12, 13]. In addition, depression increases the risk of obesity, type 2 diabetes mellitus (T2DM), cardiovascular disease (CVD), and infertility [14, 15]. Recent evidence confirmed the bidirectional link between depression with obesity and T2DM [16, 17]. Therefore, it is necessary to identify the factors related to depression as well as determine practical strategies for its management.

Recent evidence suggested that diet has an important association with mental health like depression, stress, and anxiety [18, 19]. Previous animal studies have established that high-fat diet combined with mild stress showed the most severe depression-like behavior in rats [20]; as well, it was confirmed that the high-fat diet could induce obesity through alteration of leptin levels and increasing appetite in rats under mild stress condition [21]. Healthy eating patterns are related to better mental health and the effects of certain foods on metabolic systems can play a role in the association between food and mood [22]. Adherence to the unhealthy dietary patterns, which are often high in red and processed meats, full-fat dairy products, saturated fatty acids (SFAs) and refined sugars, is directly associated with the risk of depression, low mood, and anxiety among adolescents, and adherence to the healthy dietary patterns can decrease the risk of depressive symptoms [23–26]. In addition, dietary behaviors are considered important factors in mental health [27]. Some unhealthy dietary behaviors like inadequate consumption of fruits, and vegetables as well as skipping meals are associated with feeling sad or hopeless, suicide ideation, and suicide attempts in adolescents [28–31]. A longitudinal research has reported an association between greater dietary glycemic index and the incidence of depressive symptoms [32]. Moreover, further strong value evidence has showed that avoiding processed foods and adherence to healthy diets can prevent depression [33]. As a result, it is hypothesized that many unhealthy dietary behaviors are related to the risk of depression. Generally, there are few investigations in this regard, and the relationship between dietary behaviors and depression is not fully elucidated. It seems further investigations are required to determine what extent unhealthy dietary behaviors are linked to the adolescent mental disorders. Accordingly, the present study was designed to investigate the relationship between depression and dietary behaviors in Iranian adolescent girls.

## Materials and methods

### Study population

Using a random cluster sampling method, a total of 1024 adolescent girls (12–18 years old) were randomly recruited from several schools of Mashhad and Sabzevar cities in northeastern Iran. The girls with any cardiovascular disorders, renal or hepatic failure, cancer, malabsorption, thyroid, adrenal or parathyroid diseases, eating disorders, metabolic bone disease, eating disorders and autoimmune diseases were excluded. In addition, we excluded subjects who were taking anti-inflammatory, antidiabetic or antiobesity drugs, antidepressants, vitamin D or calcium supplement use, and hormone therapy within the last 6 months. Finally, a total of 933 adolescent girls were included in statistical analysis.

### Depression assessment

Assessment of depression was performed using the 21-item BDI [34]. According to the BDI, symptoms including feelings of guilt, feelings of hopelessness, sadness, crying, sleep disturbance, fear and loss of appetite over the past 2 weeks are the items evaluating depression. Severity of depression is classified as follows: scores 0–13 refer to minimal or no depression, 14–19 illustrate mild depression, 20–28 indicate moderate depression, and 29–63 demonstrate severe depression [34]. The validity of the Persian (Farsi) questionnaire was evaluated by Ghassemzadeh et al. [35], which showed an acceptable internal consistency (Cronbach’s alpha = 0.87) and test-retest reliability (*r* = 0.74).

### Dietary intake assessment

To evaluate usual dietary intake, a validated food frequency questionnaire (FFQ) with 147 food items was utilized [36, 37]. Participants were interviewed face-to-face, and all questionnaires were completed by a trained dietitian interviewer. The frequency of food items intake during the last year was recorded by asking participants about their daily, weekly, monthly and yearly intake. Thereafter, the frequency of each food intake was converted to daily. A household-scale was utilized to convert portion sizes of consumed foods to grams. Finally, using Nutritionist IV (N-Squared Computing, Salem, OR, USA) modified for Iranian foods, the content of energy and nutrients of all coded foods and beverages were analyzed.

### Assessment of dietary behaviors

Dietary behaviors were assessed using a validated self-administered questionnaire with several domains including main meal patterns, breakfast consumption, snack consumption, meal regularity, intra-meal fluid intake, fried foods intake, spicy foods intake, ability to chewing, chewing insufficiency, and lack of teeth [38–40].

To examine the frequency of meals, participants answered the following questions: “How many main meals do you consume each day?” (one, two, or three), and “How many snacks do you consume each day?” (none, one to two, three, or more than three). To evaluate meal pattern regularity, subjects answered the following question “Do you consume your meals regularly?” (never, sometimes, almost, or always). In this regard, subjects were asked about the regularity of having breakfast with different choices as follows: never or 1 day/week, 2–4 days/week, 5–6 days/week, daily. Assessment of intra-meal fluid drinking was performed by asking questions about drinking water or fluids with meals or immediately before and after meals with choices including never, sometimes, often and always.

In addition, to assess the spicy foods intake, participants answered the following question: “How often do you have spicy foods during a week?” (never, 1–3 times, 4–6 times, 7 or more than 7 times per week). Assessment of the fried foods intake was performed based on participant’s answer to the following question: “How often do you have fried foods?” (never, 1–3 times/week, 4–6 times/week, every day). Evaluation of chewing quality was performed using the following question: How thoroughly do you chew food? (no problem, just soft foods, no food). Finally, tooth loss was evaluated by the following question: “How many teeth have you lost?” (≤ one tooth loss,” “2-4 teeth loss” and “five teeth loss”).

### Anthropometric measurements

Measuring anthropometric indices including weight, height, and waist circumference (WC) was performed using standard methods. Weight was measured using a digital scale with an accuracy of 100 g, while participants were without shoes in a minimal clothing state. Height (without shoes) was measured using a stadiometer with an accuracy of 0.1 cm. Body mass index (BMI) was calculated by the following formula: weight (kg)/height squared (m^2^). In addition, WC was measured at the narrowest level between the costal margin and iliac crest during minimal respiration in a standing position.

### Assessment of other variables

Using a demographic questionnaire, variables including age, educational level of parents, medical history, and using medications were assessed. Physical activity information was obtained by using the validated Modifiable Activity Questionnaire (MAQ). Physical activity levels were computed based on metabolic equivalent task (MET) hours per week, (MET- h/week) [41].

### Statistical analyses

Kolmogorov-Smirnov test was used to evaluate the normal distribution of variables. Data were expressed as mean ± standard deviation (SD) for normal variables. Chi-square test was used to compare the qualitative variables, and an independent t-test was utilized to compare the means of normal variables. In addition, crude and adjusted linear regression analyses were conducted to investigate the relationship between dietary behaviors and depression score. In the first model, age and energy intake were considered confounding variables. Additional adjustments were performed in the second model by controlling physical activity, menstruation, and academic degrees of parents. Moreover, additional adjustment was done for BMI in the third model. Also, in last model, the association between each dietary behavior with depression score was adjusted for other dietary behavior. The Bonferroni correction was applied to all models to adjust for multiple comparisons. The significant level was assessed in two steps: traditional cutoff of *P*-values < 0.05 and Bonferroni correction of *p*-value < 0.005 (alpha = 0.05/10 comparisons). Data were analyzed using SPSS-18 software (SPSS Inc., IL, USA).

## Results

### Demographic profile of the study population

A total of 933 participants were included in analyses and the mean ± SD for age of participants was 14.56 ± 1.52. 67.7% (*n* = 632) of participants had no or minimal depression symptoms, and 32.3% (*n* = 301) of participants were categorized with mild-to-severe depression symptoms. General characteristics of subjects across depression score categories are represented in Table 1**.** There was no significant difference across the categories of depression for age, menstruation, BMI, physical activity, energy intake, and parents’ education level (*P > 0.05*).

**Table 1:** General characteristics of study participants among different categories of Beck depression score

Variables	Depression				*P-value*^†^
	*No depression/ Minimal*	*Mild*	*Moderate*	*Severe*	
Age (year)	14.57 ± 1.53	14.54 ± 1.49	14.61 ± 1.61	14.43 ± 1.44	0.926
Menstruation, yes (%)	90.7	89.7	92.1	97.5	0.452
BMI (kg/m2)	21.36 ± 4.33	20.77 ± 3.87	21.10 ± 5.02	20.63 ± 2.93	0.386
Physical activity (MET.h/week)	45.53 ± 3.41	45.23 ± 3.59	45.78 ± 4.34	46.17 ± 4.03	0.419
Energy intake (Kcal)	2707.43 ± 824.25	2678.64 ± 844.90	2719.93 ± 879.48	2873.27 ± 844.48	0.680
Academic degree of father (%)					
Illiterate	39.4	43	49.1	37.5	0.114
Under diploma	52	51.4	45.6	45	
Academic education	8.6	15.7	5.3	17.5	
Academic degree of mother (%)					
Illiterate	48.5	49.7	53	52.5	0.203
Under diploma	44.8	46.9	44.3	35	
Academic education	6.6	3.5	2.6	12.5	

^†^Independent samples t-test for continuous variables and chi-squared test for categorical variables

There was a significant difference across categories of depression score regarding the consumption of main meal (*P < 0.001*), regular meal consumption (*P < 0.001*), breakfast consumption (*P = 0.007*), intra-meal fluid intake *(P = 0.003)*, and tooth loss (*P = 0.04*); however, no significant difference was found for snack consumption, fried or spicy foods consumption, and the ability of chewing (*P > 0.05*) (Table 2).

**Table 2:** Dietary behavior distribution of study participants among different categories of Beck depression score

Variables	Depression				*P-value *^†^
	*No depression/ Minimal*	*Mild*	*Moderate*	*Severe*	
Consumption of main meal (%)					
1 time	2.1	4.9	11.3	5.1	< 0.001
2 times	25.1	31.9	32.2	35.9	
3 times	72.8	63.2	56.5	59	
Snack consumption (%)					
None	4.4	8.3	7.3	11.1	0.213
1–2	56.7	60.4	58.2	58.3	
3–5	33.3	25	31.8	22.2	
> 5	5.6	6.2	2.7	8.3	
Regular meal consumption (%)					
never	7.4	18.8	11.6	20	< 0.001
Sometimes	38.1	36.1	53.6	42.5	
Most	31.5	31.9	22.3	25	
Always	22.9	13.2	12.5	12.5	
Rate of food chewing (%)					
Low	10.1	16.6	15.7	22.5	0.091
Average	78.9	73.8	76.5	70	
High	10.9	9.7	7.8	7.5	
Breakfast consumption (%)					
Never or 1 day	13.4	18.8	20.4	23.1	0.007
2–4 days	24.9	27.1	34.5	33.3	
5–6 days	15.1	19.4	15	17.9	
Every day	46.6	34.7	30.1	25.6	
Intra-meal fluid intake (%)					
Never	6.3	3.5	6.2	5.1	0.003
Sometimes	35	30.6	36.3	30.8	
Most	31.2	31.9	18.6	10.3	
Always	27.5	34	38.9	53.8	
Consumption of fried foods (%)					
Never	3.1	2.1	2.7	5	0.795
1–3 times	66.8	62.9	62.8	60	
4–6 times	25.7	30.1	28.3	25	
Every day	4.4	4.9	6.2	10	
Consumption of spicy foods (%)					
Never	2.7	2.8	1.8	2.6	0.075
1–3	28.4	30.6	18.6	28	
4–6	40.4	32.6	38.9	38.3	
≥7	28.4	34	40.7	31.1	
Ability of chewing (%)					
Yes	96.6	96.5	93	94.9	0.073
Only soft foods	3.1	3.5	4.3	5.1	
No	0.3	0.0	2.6	0.0	
Tooth loss (%)					
≤2	61.9	16.9	18	3.2	0.044
> 2	69.1	15.3	11	4.6	

^†^ Obtain from chi-squared test

### Dietary behaviors and depression

Crude and multivariate-adjusted linear associations for depression score across categories of diet-related behaviors are demonstrated in Table 3. There were significant inverse relationships between main meal consumption (Beta: -0.141; 95% CI: − 3.644 to − 1.000; *P = 0.001*), snack consumption (Beta: -0.100; 95% CI: − 2.400 to − 0.317; *P = 0.002*), regular meal consumption (Beta: 0.23; 95% CI: 0.13–0.42; *P = 0.001*) and food chewing (Beta: -0.152; 95% CI: − 2.279 to − 0.753; *P = 0.03*) with depression score. These associations remained significant after adjustment for confounding variables. In addition, frequency of intra-meal fluid intake (Beta: 0.096; 95% CI: 0.288 to 1.535; *P = 0.004*) and spicy foods consumption (Beta: 0.076; 95% CI: 0.098 to 1.508; *P = 0.02*) were directly associated with depression score in the crude model and some adjusted models, but not in the fully adjusted model. These significant relations were disappeared in full adjusted model. No significant association was found between fried foods consumption, chewing ability, and tooth loss with depression score (*P > 0.05*) in crude and adjusted models. After applying the Bonferroni correction, the inverse associations between main meal intake and regular meal consumption with depression were significant in crude and adjusted models, while the relations between intra-meal fluid intake and snack consumption with depression score were only remained significant in crude model (*P < 0.005*).

**Table 3:** The association between dietary behavior and depression score

Dietary habits	Beta	95%confidence interval	***P***-value^#^
***Consumption of main meal***			
Crude	−0.120	−3.054 to −0.808	0.001*
Model I	−0.127	−3.344 to − 0.797	0.001*
Model II	−0.139	−3.600 to − 0.976	0.001*
Model III	−0.141	−3.644 to −1.000	0.001*
***Snack consumption***			
Crude	−0.103	−2.211 to − 0.485	0.002*
Model I	−0.096	−2.302 to − 0.292	0.011
Model II	−0.098	−2.371 to − 0.297	0.012
Model III	−0.100	−2.400 to − 0.317	0.011
***Regular meal consumption***			
Crude	−0.117	− 1.773 to − 0.482	0.001*
Model I	−0.150	−2.221 to − 0.748	< 0.001*
Model II	− 0.151	− 2.261 to − 0.743	< 0.001*
Model III	− 0.152	− 2.279 to − 0.753	< 0.001*
***Rate of food chewing***			
Crude	− 0.071	− 2.570 to − 0.109	0.033
Model I	− 0.080	−2.979 to − 0.137	0.032
Model II	− 0.076	− 2.911 to − 0.030	0.045
Model III	− 0.081	−3.035 to − 0.125	0.033
***Breakfast consumption***			
Crude	− 0.061	− 1.011 to 0.071	0.088
Model I	− 0.073	−1.216 to 0.043	0.068
Model II	−0.066	−1.185 to 0.108	0.102
Model III	−0.065	−1.179 to 0.125	0.113
***Intra-meal fluid intake***			
Crude	0.096	0.288 to 1.535	0.004*
Model I	0.079	0.059 to 1.491	0.034
Model II	0.078	0.030 to 1.494	0.041
Model III	0.070	−0.048 to1.433	0.067
***Consumption of fried foods***			
Crude	0.018	−0.715 to1.219	0.609
Model I	0.023	−0.786 to 1.487	0.545
Model II	0.017	−0.906 to 1.423	0.663
Model III	0.020	−0.876 to 1.472	0.619
***Consumption of spicy foods***			
Crude	0.076	0.098 to 1.508	0.026
Model I	0.066	−0.095 to 1.538	0.083
Model II	0.068	−0.089 to 1.580	0.080
Model III	0.066	−0.120 to 1.567	0.093
***Ability of chewing***			
Crude	0.047	−0.726 to 4.502	0.157
Model I	0.029	−1.736 to 4.019	0.436
Model II	0.029	−1.777 to 4.039	0.445
Model III	0.032	−1.693 to 4.151	0.409
***Tooth loss***			
Crude	0.037	−0.305 to 1.103	0.266
Model I	0.011	−0.683 to 0.928	0.766
Model II	0.009	−0.716 to 0.921	0.806
Model III	0.003	−0.802 to 0.863	0.943

Model I: adjusted for age and energy intake; Model II: additionally, adjusted for physical activity, menstruation and academic degrees of parents. Model III: further adjustments for BMI and other dietary behaviour

^#^ obtained from logistic regression

* Significant after Bonferroni correction

## Discussion

Depression as a common mental disorder and an important cause of suicide among adolescents, has become a major problem [1, 2, 13]. Recent evidence suggested that lifestyle (smoking, drinking, using drugs, sedentary, and less healthy dietary behaviors) can be associated with depression and other indicators of mental health status [27, 42, 43]. Clarifying these potentially modifiable factors can increase the knowledge and ability of public health officials, clinicians and patients to manage/improve depression. Therefore, we conducted a large cross-sectional study to investigate the association between adherence to dietary behaviors and depression score.

The present cross-sectional study showed that the frequency of main meal consumption, snack consumption, regular meal consumption and rate of food chewing were inversely associated with depression score in Iranian adolescent girls. There were direct significant relations between Intra-meal fluid intake and spicy food consumption with depression score in crude and some adjusted models, but not in the fully adjusted model. However, there was no significant relationship between breakfast consumption, fried food intake, ability of chewing and tooth loss with depression score in crude or adjusted models.

We mentioned that there was an inverse relationship between the frequency of main meal consumption, snack consumption, and regularity in meal consumption with depression score. People who feel depressed, hopeless, or other mental health disorders may skip main meals and snacks because of their poor appetite; on the other hand, skipping meals reduces calorie intake, which can increase depressed mood and anhedonia [44]. There are few investigations in this field. In line with our findings, a case-control study showed that the average dietary behavior scores of “eating meals at regular times” and “eating an adequate amount of meals” are significantly lower in students with depression compared to the healthy controls [45].

Our study was not found a significant relationship between breakfast consumption and depression score in adolescents, while some previous studies have reported that adolescents may not consume their breakfast due to their irregular sleep pattern [45]. Fulkerson et al. [28] and Arbour-Nicitopoulos et al. [31] showed the association between inadequate intake of breakfast with risk of depressive symptoms in adolescents. Moreover, the study of Michael et al. [27] demonstrated that there is a relationship between skipping breakfast and elevated risk of mental disorders in high school students. On the other hand, irregular meals or skipping breakfast may lead to obesity in children and adolescents; and obesity known as a risk factor for depressive symptoms in adolescence [46]. Correcting this issue requires multilevel efforts, and the family (by regulating sleep time and planning mealtimes of adolescents) and school (codification of a school breakfast program) can play a critical role.

In addition, an inverse relationship was obtained between the rate of food chewing and depression score; but not chewing ability as well as tooth loss. A study conducted by Aljameel et al. [47] showed that there is an association between depression and chewing difficulty in adults, independent of demographic and socio-economic characteristics. The study of Kimora et al. [48] illustrated a significant association between low chewing ability (assessment of chewing ability using color-changeable chewing gum) and depression in elderly people. The logical reasons for these minor discrepancies can be the differences in design, age of subjects and methodology of studies. Oral health can negatively impact the daily life activities and psychological wellbeing of individuals [49]. Interestingly, a study evaluating the emotional effects of tooth loss and its association with depression indicated that the group which had difficulties accepting tooth loss showed more depressive symptoms than the group with no difficulties [50]. On the other hand, depression can reduce oral health and chewing ability by decreasing the motivation towards oral health-related behaviors, like oral hygiene and healthy dietary habits [51, 52], as well as reducing the amount of saliva or the immune response system dysregulation [53].

The present study revealed that there is a direct association between consumption of spicy foods and depression score in crude model. Based on our finding, consumption of spicy foods less than 7 times per week has no association with depression. Therefore, this result must be interpreted with caution. There are very few studies evaluating the association between spicy food consumption and depression. A case-control study showed that the average dietary habit score of “avoiding eating spicy foods” was significantly lower in patients with depression compared with the healthy female adolescents [46]. The exact relationship and mechanisms in this field are still unclear.

No significant relationship between fried foods consumption and depression score was found. Contrary to our results, a cross-sectional study demonstrated a direct association between frequency of fried food consumption and depression score in women [54]. The differences of the studies in methodology, and race as well as the age of participants, may explain the discrepancy in the findings. Early evidence showed that fried foods consumption, which is a part of the western dietary pattern, can increase the risk of obesity, inflammation and oxidative stress [55–58], that these are linked to the risk of depressive symptoms and other mental disorders [6, 7, 9, 15].

Finally, we found a significant association between intra-meal fluid intake and depression in crude and some adjusted models, but not in the fully adjusted model. Based on our knowledge, there was no similar study evaluating the association between intra-meal fluid intake and depression. We mentioned that obesity could contribute to the development of depressive symptoms [15, 16]. A cross-sectional study demonstrated that consuming 1–2 glasses of fluids between meals increased the chance of general obesity [59]. It should be noted that we don’t ask subjects about their type of beverages. Water is the main beverage consumed with the meal in the Iranian culture [60]. Studies showed a positive association between sugar-sweetened beverages intake and obesity [61, 62]. Moreover, the replacement of sugar-sweetened beverages with water can decrease weight gain [63, 64].

Another issue may be related to depression in female adolescents is their body weight and self-esteem [33]. It has demonstrated that male gender and lower BMI were associated with better body esteem in adolescents [65]. On the other hand, body dissatisfaction can lead to adverse mental problems like bulimic and depressive symptoms in girl adolescents [66].

Our study has some important strength points. This study was conducted in a large sample and is one of the first studies to examine the relationship between dietary behavior and depression score in adolescent girls. To avoid misleading conclusions in analysis and interpretation of data, we conducted several adjustment models for confounding factors to depression. Nevertheless, our findings should be interpreted in the light of some potential limitations. Cross-sectional design should be considered as the major limitation of the present study; and cannot therefore assume a causal relationship. Dietary intake data was collected using an FFQ which may lead to misclassification and over/underestimation. Moreover, depression score was obtained by Beck’s Depression Inventory; while the depression diagnosis based on clinical procedures could result in more reliable results. In addition, this study included only adolescent females; therefore, In future studies male gender should be also included.

## Conclusions

In conclusion, frequency of main meal consumption, snack consumption, regular meal consumption, and rate of food chewing were inversely associated with depression score. We were unable to confirm significant association between breakfast consumption, fried food intake, ability of chewing and tooth loss with depression score. Prospective studies are necessary to clarify the relationship between specific dietary behaviors and the risk of depression.

## Data Availability

The data of the present study is available from the corresponding author on reasonable request.

## Abbreviations

BDI: Beck depression inventory
BMI: Body mass index
CVD: Cardiovascular disease
FFQ: Food frequency questionnaire
MET: The metabolic equivalent of task
OR: Odds ratio
SD: Standard deviation
SFA: Saturated fatty acid
T2DM: Type 2 diabetes mellitus
WC: Waist circumference
